# Chronic Lung Disease: A Study Based on the National Database of Specified Chronic Pediatric Diseases of Japan

**DOI:** 10.1111/ped.70514

**Published:** 2026-08-13

**Authors:** Fumihiko Namba

**Affiliations:** ^1^ Department of Pediatrics, Saitama Medical Center Saitama Medical University Saitama Japan

**Keywords:** bronchopulmonary dysplasia, chronic lung disease, low birth weight, prematurity, specific pediatric chronic diseases

## Abstract

**Background:**

Chronic lung disease (CLD), commonly recognized as bronchopulmonary dysplasia and primarily associated with prematurity and low birth weight, is designated as a specified chronic pediatric disease in Japan. However, the characteristics of children registered in the National Database of Specified Chronic Pediatric Diseases have not been fully elucidated.

**Methods:**

With approval from the Ethics Committee of Saitama Medical Center, Saitama Medical University, and the Third‐Party Data Provision Office for Patients with Intractable Diseases, we analyzed data from Medical Certificates of Specific Chronic Pediatric Diseases for patients with CLD registered between January 2015 and March 2023.

**Results:**

A total of 1737 patients with CLD were identified. Extremely preterm infants and term infants accounted for 47% and 24% of cases, respectively, with marked regional variation in gestational age distribution. Among 410 registered term infants, neurological disorders (43%) and chromosomal abnormalities (19%) were the most common underlying conditions. With increasing age, the proportion of extremely preterm infants decreased, whereas that of term infants increased. In a subgroup analysis of 805 extremely preterm infants, invasive mechanical ventilation at 36 weeks' postmenstrual age was independently associated with persistent registration within the chronic disease support program (odds ratio 3.16, 95% CI 1.81–5.52; *p* < 0.001).

**Conclusions:**

This nationwide registry study demonstrates that registry‐defined CLD in Japan represents a heterogeneous population, including a substantial number of term infants with diverse comorbidities. Among extremely preterm infants, the need for invasive respiratory support at 36 weeks' postmenstrual age was associated with persistent registration within the chronic disease support program, underscoring the importance of optimizing long‐term management strategies.

## 1 Introduction

Chronic lung disease of infancy (CLD), also known as bronchopulmonary dysplasia (BPD), is a prevalent condition in preterm infants. In 1967, Northway was the first to describe BPD as a pulmonary sequela of long‐term mechanical ventilation and oxygen therapy in premature infants with severe respiratory distress syndrome [1]. CLD/BPD is a chronic respiratory disease primarily marked by retardation and arrest of pulmonary vascular and alveolar growth [2]. Despite the advent of modern perinatal care, including antenatal corticosteroids, exogenous surfactant replacement therapy, and lung‐protective strategies with mechanical ventilation, which have culminated in the amelioration of survival rates for very premature infants, CLD/BPD persists as the most prevalent complication of extremely preterm birth. CLD/BPD poses a significant challenge not only in the acute management of preterm infants but also leads to long‐term respiratory complications, including increased rates of rehospitalization for respiratory problems in childhood and adolescence, as well as a lifelong decline in respiratory function. Furthermore, it has been demonstrated to exert a detrimental influence on the neurodevelopmental outcomes of preterm infants [3].

In Japan, pediatric chronic diseases classification is delineated by a comprehensive system that encompasses 788 distinct diseases, grouped into 16 categories, 278 subcategories, and 845 sub‐subcategories as of November 1, 2021. Japan's Medical Aid Program for Chronic Pediatric Diseases of Specified Categories was first established in Japan in 1974, and it was solidified into law and made a formal national measure in 2005. Patients suffering from specific pediatric chronic diseases may be eligible for government support and welfare in several ways. First, diagnosis and medical treatment at designated hospitals and by designated physicians are required to enable patients to receive optimal treatment. Second, the copayment rate for medical expenses is reduced to 20%, and a maximum is set depending on household income. Third, the analysis of specific pediatric chronic diseases from different perspectives is continuously being promoted. In addition to the aforementioned three aspects, various measures are being implemented to provide ongoing support for patients with specific pediatric chronic diseases as they mature [4].

According to the Information Center for Specific Pediatric Chronic Diseases, CLD is diagnosed based on the presence of a primary clinical manifestation, namely, the requirement of oxygen supplementation that exceeds 21% within 28 days of birth, attributable to lung or airway disorders. This requirement persists daily for more than several days, without any transient nature. Furthermore, abnormalities in the lungs detected by radiological examination and oxygen supplementation exceeding 21% or positive pressure support at a postmenstrual age (PMA) of 36 weeks are used as the reference for determining severity and disease type. CLD patients who require respiratory management, including mechanical ventilation, tracheostomy, and nasal airway oxygen therapy or total parenteral nutrition, are registered as having a specific pediatric chronic disease.

To the best of our knowledge, there have been no analyses employing the data of CLD patients from the National Database of Specified Chronic Pediatric Diseases of Japan to date. Therefore, this study aimed to evaluate the characteristics of children registered with CLD using the National Database of Specified Chronic Pediatric Diseases of Japan.

## 2 Methods

### 2.1 Setting and Participants

This retrospective study included children registered as CLD patients in the National Database of Specified Chronic Pediatric Diseases of Japan between January 1, 2015 and March 31, 2023. Data were obtained from Medical Certificates of Specific Chronic Pediatric Diseases with approval from the Research Ethics Committee of Saitama Medical Center, Saitama Medical University (approval number: 2023‐012), as well as the Third‐Party Data Provision Office for Patients with Intractable Diseases and its specialized committee. Because this was a retrospective study, the requirement for informed consent was waived. In the present study, the author acknowledges that the statistics are derived from the National Database of Specified Chronic Pediatric Diseases of Japan and were independently created and processed by the author. These data differ from the statistics created and published by the Ministry of Health, Labour and Welfare. During the preparation of this work, DeepL Write was used in order to improve readability and language. After using this tool, the content was reviewed and edited as needed and the author takes full responsibility for the content of the publication.

### 2.2 Perinatal Data and Measurements

Perinatal and neonatal data were retrospectively collected from Medical Certificates of Specific Chronic Pediatric Diseases. Gestational age was determined in the following order: obstetric examination via ultrasonography, obstetric history‐taking based on the mother's last menstrual period, and postnatal physical examination of neonates. Histological chorioamnionitis was defined as stage 1 or higher per the Blanc classification [5]. Diffuse chorioamniotic hemosiderosis was defined as diffuse iron stain–positive, bile stain–negative pigment deposition in the chorioamniotic layers of the chorionic plate and/or membranes [6]. Small for gestational age was defined as a birth weight below the 10th percentile of the expected value for the gestational age [7]. Hyper‐IgM was defined as serum IgM levels ≥ 30 mg/dL [8]. Persistent registration was defined as continued registration in the National Database of Specified Chronic Pediatric Diseases of Japan beyond 2 years of age.

### 2.3 Statistical Analysis

Between‐group baseline feature and outcome comparisons were performed using Fisher's exact test for categorical variables and the Mann–Whitney nonparametric *U* test for continuous variables. The adjusted odds ratio and 95% confidence interval of risk factors for persistent registration within the chronic disease support program were determined using logistic regression analyses adjusted for gestational age, sex, chorioamnionitis, diffuse chorioamniotic hemosiderosis, hyper‐IgM (≥ 30 mg/dL), congenital anomaly, and the use of invasive mechanical ventilation and oxygen (FiO_2_ ≥ 0.30) at 36 weeks PMA. Covariates were selected a priori based on previously reported risk factors for moderate/severe BPD. Variance inflation factors were calculated and no evidence of significant multicollinearity was observed (all variance inflation factors < 2). All statistical data were analyzed using EZR (Saitama Medical Center, Jichi Medical University, Saitama, Japan) [9], a graphical user interface for R (version 4.3.0; R Foundation for Statistical Computing, Vienna, Austria). Continuous data are presented as medians with their corresponding interquartile ranges, while categorical data presented as frequencies and percentages. Two‐sided *p*‐values of < 0.05 were considered statistically significant.

## 3 Results

### 3.1 Patient Characteristics

**FIGURE 1:**
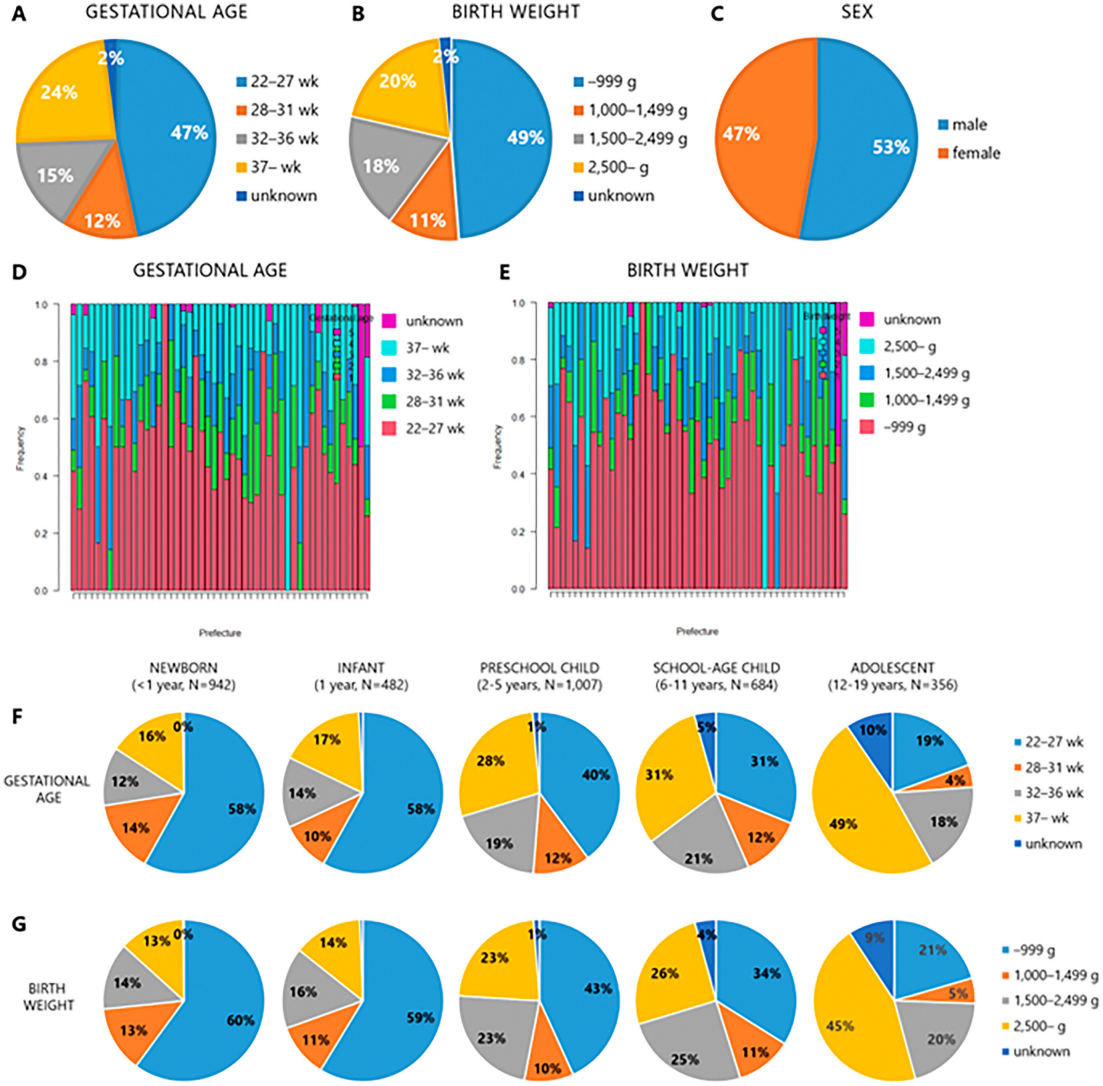
(A–C) Perinatal characteristics of eligible children. (A) Gestational age. (B) Birth weight. (C) Sex. (D, E) Distribution of eligible children across prefectures. (D) gestational age. (E) Birth weight. (F, G) Distribution of eligible children in the different age groups. (F) Gestational age. (G) Birth weight.

In total, 1737 children registered as CLD patients in the National Database of Specified Chronic Pediatric Diseases of Japan between January 2015 and March 2023 were evaluated. The gestational age, birth weight, and sex distributions are presented in Figure 1A–C. Among the registry‐defined CLD patients in this cohort, 47% were extremely preterm infants (< 28 weeks of gestation), and 49% had extremely low birth weight (< 1000 g). In contrast, 24% and 20% were term infants (≥ 37 weeks) and infants with a normal birth weight (≥ 3000 g), respectively. Table 1 shows the significant differences in the perinatal characteristics of the two groups of infants in this cohort: those born extremely preterm and those born at term. Characteristics of infants born at 28–36 weeks' gestation are presented in Table S1. The male‐to‐female ratio was 53:47. Figure 1D,E shows the gestational age and birth weight distributions of patients registered under the CLD category of the Specific Chronic Pediatric Diseases Program by prefecture in Japan, highlighting regional variation in these features.

**TABLE 1:** Perinatal characteristics and respiratory management in extremely preterm and term infants with registry‐defined CLD.

	Extremely preterm infants (*n* = 809)	Term infants (*n* = 410)	*p*
Perinatal characteristics			
Gestational age, week	25 (23, 26)	38 (37, 40)	< 0.001
Birth weight, g	646 (524, 778)	2719 (2409,3078)	< 0.001
Male	429/809 (53.0)	217/410 (52.9)	1.000
Histological chorioamnionitis	395/768 (51.4)	14/349 (4.0)	< 0.001
Serum IgM levels, mg/dL	7.0 (4.0, 25.0)	10.6 (7.0, 33.5)	< 0.001
Respiratory distress at delivery	783/798 (98.1)	301/386 (78.0)	< 0.001
Onset of respiratory distress, day	0 (0, 0)	0 (0, 1)	< 0.001
Congenital anomaly	101/766 (13.2)	216/381 (56.7)	< 0.001
Respiratory management			
Invasive ventilation, Day 28	694/782 (88.7)	157/374 (42.0)	< 0.001
Invasive ventilation, 36 weeks of PMA	292/773 (37.7)	N/A	
Oxygen use, Day 28	730/755 (96.7)	238/362 (65.7)	< 0.001
Oxygen use, 36 weeks of PMA	708/765 (92.5)	N/A	
FiO_2_, Day 28	0.30 (0.30, 0.40)	0.25 (0.21, 0.30)	< 0.001
FiO_2_, 36 weeks of PMA	0.30 (0.25, 0.30)	N/A	

*Note:* Data are presented as median (interquartile range) or number (%).

Abbreviations: CLD, chronic lung disease; FiO_2_, fraction of inspired oxygen; PMA, postmenstrual age.

**FIGURE 2:**
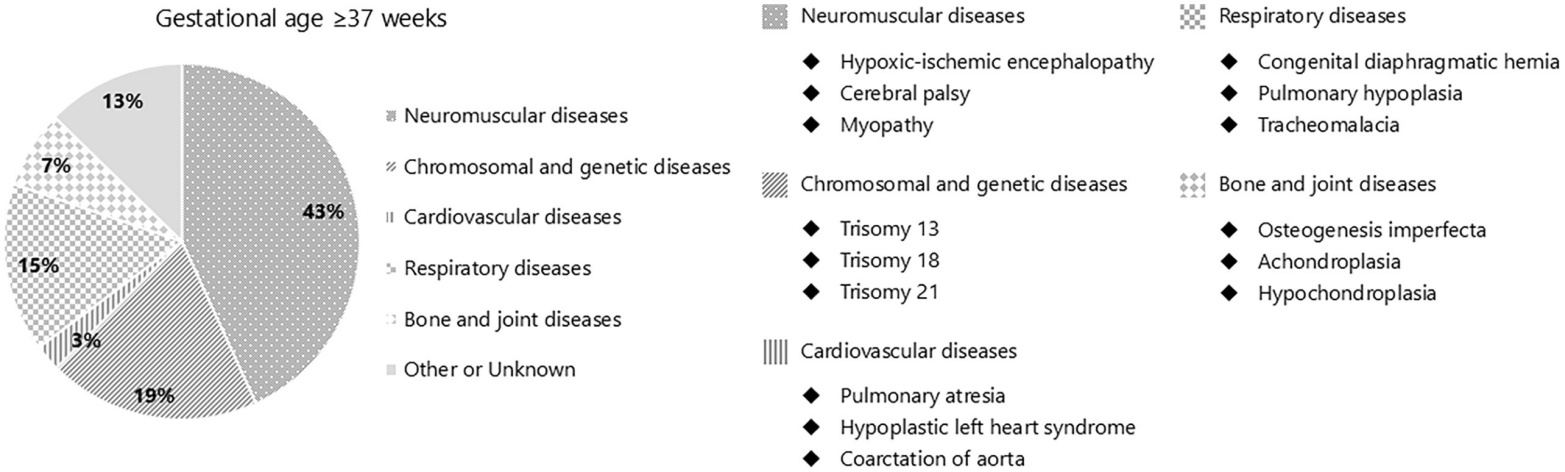
Underlying diseases in eligible children born at term (≥ 37 weeks of gestation).

Subsequently, the gestational age and birth weight distributions were analyzed by age group. As patient age increased, the proportion of children born extremely preterm and/or with extremely low birth weight decreased from 60% to 20%, while the proportion of those born at term and/or with normal birth weight increased from 15% to 50% (Figure 1F,G). Among the 410 prematurity‐associated CLD patients who were born at term (≥ 37 weeks of gestation), the most common underlying diseases were neuromuscular disorders, including hypoxic–ischemic encephalopathy, cerebral palsy, and myopathy, accounting for 43% (176/410). Chromosomal and genetic disorders, such as trisomy 13, 18, and 21, were the second most frequent (78/410, 19%), followed by respiratory diseases, including congenital diaphragmatic hernia, pulmonary hypoplasia, and tracheomalacia (62/410, 15%) (Figure 2).

### 3.2 Factors Associated With Persistent Registration Within the Chronic Disease Support Program

After four patients were excluded from the registration‐status analysis due to unavailable follow‐up information, the 805 registry‐defined CLD patients born before 28 gestational weeks were categorized into the early registration discontinuation group (*n* = 474) and the persistent registration group (registration beyond 2 years of age) (*n* = 331). Compared with the early registration discontinuation group, the persistent registration group had a lower incidence of histological chorioamnionitis and a higher rate of invasive mechanical ventilation at 36 weeks PMA. The other perinatal characteristics and respiratory management parameters, including gestational age, sex distribution, presence of diffuse chorioamniotic hemosiderosis, congenital anomalies, serum IgM levels, oxygen use, and FiO_2_ at 36 weeks PMA, did not differ significantly between the two groups (Table 2). The multivariate logistic regression analysis demonstrated that, among registry‐defined CLD patients born at < 28 weeks of gestation, the use of invasive mechanical ventilation at 36 weeks PMA was the factor independently associated with persistent registration within the chronic disease support program (odds ratio: 3.16; 95% confidence interval: 1.81–5.52; *p* < 0.001) (Table 3).

**TABLE 2:** Characteristics of extremely preterm infants with CLD by registration status.

	Early registration discontinuation group (*n* = 474)	Persistent registration group (*n* = 331)	*p*
Perinatal characteristics			
Gestational age, week	25 (23, 26)	25 (23, 26)	0.221
Birth weight, g	645 (524, 786)	650 (523, 778)	0.915
Male	241 (50.8)	183 (55.3)	0.223
Histological chorioamnionitis	266 (57.1)	132 (43.3)	< 0.001
Diffuse chorioamniotic hemosiderosis	037 (08.6)	019 (06.7)	0.395
Serum IgM levels, mg/dL	8 (4, 24)	7 (4, 35)	0.820
Congenital anomaly	044 (10.0)	055 (17.7)	0.002
Respiratory management			
Invasive ventilation, Day 28	398 (86.1)	284 (92.2)	0.011
Invasive ventilation, 36 weeks of PMA	122 (26.5)	162 (53.3)	< 0.001
Oxygen use, Day 28	441 (97.4)	285 (96.3)	0.426
Oxygen use, 36 weeks of PMA	428 (93.9)	271 (90.9)	0.152
FiO_2_, Day 28	30 (30, 40)	30 (30, 40)	0.067
FiO_2_, 36 weeks of PMA	30 (25, 33)	30 (25, 33)	0.897

*Note:* Data are presented as median (interquartile range) or number (%).

Abbreviations: CLD, chronic lung disease; FiO_2_, fraction of inspired oxygen; PMA, postmenstrual age.

**TABLE 3:** Risk factors for persistent registration in extremely preterm infants with CLD.

	Crude ORs	*p*	Adjusted ORs	*p*
Gestational age, week	1.06 (0.97, 1.17)	0.221	1.00 (0.83, 1.21)	0.973
Male	0.84 (0.63, 1.11)	0.214	0.96 (0.56, 1.65)	0.880
Histological chorioamnionitis	0.57 (0.43, 0.77)	< 0.001	1.07 (0.60, 1.93)	0.811
Diffuse chorioamniotic hemosiderosis	0.76 (0.43, 1.35)	0.353	0.56 (0.20, 1.58)	0.272
Serum IgM ≥ 30 mg/dL	1.48 (0.88, 2.47)	0.138	1.27 (0.66, 2.46)	0.471
Congenital anomaly	1.95 (1.27, 2.99)	0.002	0.96 (0.37, 2.45)	0.927
Invasive ventilation, 36 weeks of PMA	3.18 (2.34, 4.32)	< 0.001	3.16 (1.81, 5.52)	< 0.001
FiO_2_ ≥ 0.30, 36 weeks of PMA	0.98 (0.68, 1.41)	0.925	0.65 (0.38, 1.13)	0.126

*Note:* Data are presented as odds ratio (95% confidence interval).

Abbreviations: CLD, chronic lung disease; FiO_2_, fraction of inspired oxygen; OR, odds ratio; PMA, postmenstrual age.

## 4 Discussion

In this study, we examined the characteristics of children registered as CLD patients in the National Database of Specified Chronic Pediatric Diseases of Japan. Prematurity‐associated CLD/BPD is one of the most common and serious complications affecting extremely preterm infants [10], who often require prolonged neonatal intensive care unit hospitalization. After discharge, many patients require home oxygen therapy and often experience rehospitalization due to reactive airway disease and increased susceptibility to viral infections [11]. CLD/BPD engenders not only long‐term respiratory problems but also lifelong morbidity in preterm infants, including neurodevelopmental impairment [12, 13]. A study of Japanese data on infants born between 22 and 27 weeks of gestation and registered with the Neonatal Research Network of Japan revealed a higher incidence of CLD/BPD in the most immature infants. In 2016, the incidence of CLD/BPD was 134/173 (77.5%) in infants born at 22–23 weeks, 224/381 (58.8%) in those born at 24–25 weeks, and 226/568 (39.8%) in those born at 26–27 weeks of gestation. These data suggest that CLD/BPD is prevalent in extremely preterm infants [14]. In this study, however, only 47% of children registered as CLD patients in the National Database of Specified Chronic Pediatric Diseases of Japan were extremely preterm infants (born at < 28 weeks of gestation), and 24% were term infants born at term (≥ 37 weeks of gestation). Figure 1D shows that the gestational age distribution in this cohort varies by prefecture, indicating that in some prefectures, term infants who require respiratory support due to specific conditions or diseases, including neuromuscular, chromosomal, and genetic diseases, were registered in the National Database of Specified Chronic Pediatric Diseases of Japan as CLD. One possible explanation is that some term infants requiring long‐term respiratory support were registered under the CLD category because the current framework of the Specific Chronic Pediatric Diseases Program does not provide a separate respiratory category for children with neuromuscular, genetic, or congenital disorders. Since public financial assistance for medical expenses is provided to children registered in the database, the definition of CLD should be clarified and regional disparities eliminated.

This cohort included two categories of subjects: extremely preterm infants with CLD/BPD due to immaturity and term infants who required respiratory support due to other diseases. For further analyses, we excluded infants born at ≥ 28 weeks of gestation. In this study, the percentage of registry‐defined CLD infants born before 28 weeks of gestation decreased from 58% at less than 2 years of age to 40% at 2–5 years of age. Some studies have assessed the age at which children with prematurity‐associated CLD/BPD are weaned from home supplemental oxygen use. Yeh reported that the median weaning age was 12.5 months (95% confidence interval: 10.9–14.2), though it varied based on the oxygen flow rate at NICU discharge [15]. Grajangdara et al. demonstrated that, of 40 infants treated with HOT, 18 (45%) were successfully weaned from oxygen within 12 months, with a median corrected age of oxygen withdrawal of 13.8 months (interquartile range: 8.5–22.1) [16]. The natural history of prematurity‐associated CLD/BPD patients who require HOT may explain why most children in this cohort changed from being extremely preterm infants with CLD to being late preterm or term infants requiring respiratory support due to other diseases at age 2–5.

We categorized registry‐defined CLD patients into early registration discontinuation and persistent registration groups based on their duration of registration in the National Database of Specified Chronic Pediatric Diseases of Japan. Thereafter, we tried to identify factors associated with persistent registration within the chronic disease support program in extremely preterm infants with the most severe CLD cases registered in this database. In a multivariate logistic regression analysis, invasive ventilation at 36 weeks PMA was the only factor independently associated with a persistent registration within the chronic disease support program in registered extremely preterm infants. Meanwhile, other risk factors for moderate/severe prematurity‐associated CLD/BPD, including gestational age, sex, existence of intrauterine infection/inflammation, and oxygen requirement at 36 weeks PMA, were not independently associated with a persistent registration within the chronic disease support program [17]. Jensen et al. assessed infants born before 32 weeks of gestation at 18 centers in the US. They aimed to determine which CLD/BPD definition (which varies in how it defines disease severity based on the level of respiratory support and supplemental oxygen administered at 36 weeks PMA) best predicts death or serious respiratory morbidity through 18–26 months of corrected age. They concluded that the CLD/BPD definition that best predicts early childhood morbidity categorizes disease severity based on the mode of respiratory support administered at 36 weeks PMA, regardless of supplemental oxygen use [18]. Our results are consistent with their conclusion, suggesting that an oxygen requirement of > 30% at 36 weeks PMA, which was one of the indices in the prematurity‐associated CLD/BPD definition proposed at a consensus conference sponsored by the National Institutes of Health in 2001, will no longer be able to predict long‐term pulmonary prognosis or be used to classify CLD/BPD severity.

Our study has several limitations. First, the study cohort consisted of two categories of children: those born extremely prematurely who developed prematurity‐associated CLD/BPD and those born at term who required respiratory support due to other diseases, such as neuromuscular, chromosomal, or genetic diseases. Furthermore, although the prognostic analysis was restricted to infants born before 28 weeks' gestation, not all infants in this subgroup necessarily fulfilled the strict clinical definition of prematurity‐associated CLD/BPD because some had congenital anomalies or chromosomal disorders. To address this issue, congenital anomalies were included as a covariate in the multivariable analysis; however, residual confounding cannot be excluded. Second, we acknowledge that respiratory support, monitoring device use, and eligibility for government support and welfare extensions may depend, in part, on family and prescribing care provider preferences. Third, there is a possibility of bias in our study results due to the incomplete follow‐up of some eligible infants.

The current framework of the Specific Chronic Pediatric Diseases Program allows registration of patients with CLD irrespective of gestational age. While this approach may facilitate access to medical and financial support for children with chronic respiratory insufficiency, it also results in a heterogeneous patient population within the registry‐defined CLD category. These findings suggest that the current registry category includes children whose respiratory disorders differ substantially from prematurity‐associated CLD/BPD, which may affect the interpretation of epidemiological analyses.

In 2023, a revised Japanese classification system for prematurity‐associated CLD/BPD was proposed [19]; however, the diagnostic criteria themselves were not substantially changed and continue to be based on the traditional Japanese definition established by the Ministry of Health, Labour and Welfare research group [20]. According to this framework, CLD/BPD is fundamentally a disorder associated with prematurity and impaired lung development. Therefore, term infants with chronic respiratory insufficiency secondary to neuromuscular, genetic, congenital, or other disorders should not generally be regarded as having prematurity‐associated CLD/BPD. Although these children require ongoing medical, social, and financial support, consideration should be given to providing such support through disease categories distinct from prematurity‐associated CLD/BPD. Future discussions regarding the Specific Chronic Pediatric Diseases Program should consider how the revised classification system can be applied while maintaining consistency with the established diagnostic criteria for prematurity‐associated CLD/BPD.

In conclusion, this nationwide registry analysis revealed that children registered under the CLD category of the Specific Chronic Pediatric Diseases Program in Japan are a heterogeneous population that includes a substantial proportion of term and normal birth weight infants with diverse underlying conditions. Among extremely preterm infants, persistent registration within the chronic disease support program was strongly associated with the need for invasive respiratory support at 36 weeks postmenstrual age. These findings underscore the importance of tailored long‐term management strategies and highlight the need for further research to clarify regional differences and optimize care pathways for children registered under the CLD category of the Specific Chronic Pediatric Diseases Program.

## Author Contributions

F.N. was responsible for the conceptualization, design, acquisition of funding for the study, and drafting the article. All authors gave approval of the final version.

## Funding

This research is supported by the Research Program on Rare and Intractable Diseases, Health, Labour and Welfare Sciences Research Grants (23FC0104).

## Conflicts of Interest

The author declares no conflicts of interest.

## Supporting information


**Table S1:** Perinatal characteristics and respiratory management in infants with registry‐defined CLD.

## Data Availability

Research data are not shared.
